# A Novel *aadA* Aminoglycoside Resistance Gene in Bovine and Porcine Pathogens

**DOI:** 10.1128/mSphere.00568-17

**Published:** 2018-02-28

**Authors:** Andrew Cameron, Cassidy L. Klima, Reuben Ha, Robert J. Gruninger, Rahat Zaheer, Tim A. McAllister

**Affiliations:** a Agriculture and Agri-Food Canada, Lethbridge Research and Development Centre, Lethbridge, Alberta, Canada; b Faculty of Veterinary Medicine, University of Calgary, Calgary, Alberta, Canada; c Feedlot Health Management Services, Okotoks, Alberta, Canada; Escola Paulista de Medicina/Universidade Federal de Sao Paulo

**Keywords:** *Histophilus somni*, *Mannheimia haemolytica*, *Pasteurella multocida*, aminoglycoside-modifying enzymes, aminoglycosides, antibiotic resistance, bovine respiratory disease, cosmid library, integrative and conjugative element, spectinomycin

## Abstract

A novel variant of the AAD(3″) class of aminoglycoside-modifying enzymes was discovered in fatal bovine respiratory disease-associated pathogens *Pasteurella multocida* and *Histophilus somni*. The *aadA31* gene encodes a spectinomycin/streptomycin adenylyltransferase and was located in a variant of the integrative and conjugative element ICE*Mh1*, a mobile genetic element transmissible among members of the family *Pasteurellaceae*. The gene was also detected in *Mannheimia haemolytica* from a case of porcine pneumonia and in *Moraxella bovoculi* from a case of keratoconjunctivitis.

**IMPORTANCE** Aminoglycosides are important antimicrobials used worldwide for prophylaxis and/or therapy in multiple production animal species. The emergence of new resistance genes jeopardizes current pathogen detection and treatment methods. The risk of resistance gene transfer to other animal and human pathogens is elevated when resistance genes are carried by mobile genetic elements. This study identified a new variant of a spectinomycin/streptomycin resistance gene harbored in a self-transmissible mobile element. The gene was also present in four different bovine pathogen species.

## Abstract (precis)

Aminoglycosides are important antimicrobials used worldwide for prophylaxis and/or therapy in multiple production animal species. The emergence of new resistance genes jeopardizes current pathogen detection and treatment methods. The risk of resistance gene transfer to other animal and human pathogens is elevated when resistance genes are carried by mobile genetic elements. This study identified a new variant of a spectinomycin/streptomycin resistance gene harbored in a self-transmissible mobile element. The gene was also present in four different bovine pathogen species.

## OBSERVATION

In the last decade, multidrug-resistant (MDR) bacterial isolates of the bovine respiratory disease (BRD) complex have emerged that harbor mobile genetic elements (MGEs) containing genes that confer resistance to many veterinary antimicrobials (1). Widespread resistance in BRD bacterial pathogens—primarily the gammaproteobacterial *Pasteurellaceae* family members *Mannheimia haemolytica*, *Pasteurella multocida*, and *Histophilus somni* (2)—would be devastating to the beef and dairy industries (3). Discovered in the 1940s, aminoglycosides are broad-spectrum antimicrobials; veterinary formulations may include spectinomycin, dihydrostreptomycin, gentamicin, hygromycin B, and neomycin (4, 5). Collectively, they are the fifth-most-used drug class, accounting for 2%, by weight (344,120 kg), of the veterinary antimicrobials sold (5). Notwithstanding off-label usage, spectinomycin has been withdrawn from therapeutic BRD usage in Canada but continues to be used in the United States and is approved in both countries for in-feed prophylaxis for chickens and swine, often in combination with lincomycin (FDA Center for Veterinary Medicine, Health Canada Drug Product Database). Aminoglycoside resistance may involve 16S rRNA target modification or methylation, efflux, decreased permeability, and enzymatic inactivation by aminoglycoside-modifying enzymes (AMEs) (4, 6). The AMEs include *N*-acetyltransferases, *O*-phosphotransferases, and *O*-adenylyltransferases (AADs/ANTs). Inactivation of spectinomycin is commonly via adenylylation, and AAD(3″) class enzymes confer resistance to both spectinomycin and streptomycin, despite structural dissimilarities between the two drugs. The AAD(3″) enzymes adenylylate streptomycin at the 3″-OH position of the streptomycin glucosamine ring and adenylylate spectinomycin at the 9-OH position of the spectinomycin actinamine ring (4, 6, 7). The favored gene nomenclature is *aadA*, and versions/alleles are numbered (8).

We functionally identified the mechanism of spectinomycin resistance in two pathogens from diseased lung tissue from separate fatal cases of BRD in Alberta, Canada, isolated and identified to the species level as previously described (9). Unable to detect known AMEs via PCR, we constructed genomic DNA cosmid libraries to functionally screen for the spectinomycin antimicrobial resistance gene (ARG) and to examine the ARG context. Briefly, DNA was purified from *P. multocida* strain PM13 and *H. somni* strain HS31 via phenol-chloroform extraction (9). Two large (~35-kb)-insert libraries were constructed in *Escherichia coli* EPI300-T1R in accordance with the instructions for the pCC1FOS CopyControl fosmid library production kit (Lucigen), with modification (10). Transformants were plated onto Luria-Bertani (LB) agar containing chloramphenicol (12.5 µg/ml), and harvested colonies (~14,000/library) were pooled. The number of clones ensuring a 99% probability that a given sequence of a typical *P. multocida* genome (~2.3 Mb) will be contained within a 35-kb insert cosmid library was determined to be ~300.3 colonies (11).

Spectinomycin resistance screening was performed by separately inoculating 5 ml of LB broth with the pooled cosmid libraries, which were grown for ~2 h at 37°C with shaking at 200 rpm. Cultures were standardized to an optical density at 600 nm of 0.1, and a 10-fold dilution series was plated on LB agar with or without spectinomycin (50 µg/ml; Millipore Sigma). Empty vector EPI300 was used as a comparator for false-positive enumeration, but no false-positive colonies were observed after 24 h of growth at 37°C. For each library, 12 spectinomycin-resistant cosmids were extracted (EZ-10 Plasmid DNA Miniprep kit; BioBasic) and analyzed for uniqueness by EcoRI/BamHI restriction digestion (New England Biolabs) (not shown). DNA from three unique cosmids from both HS31 and PM13 libraries was prepared for Illumina MiSeq PE250 sequencing (Génome Québec). Reads (~175,000/cosmid) and the Illumina adaptor were trimmed with Trimmomatic 0.36 (criteria: phred33, LEADING:3 TRAILING:3 SLIDINGWINDOW:4:15 MINLEN:36; Reads surviving = >98%) (12), assembled with SPAdes 3.10.1 (13), and annotated with PROKKA (14) by using default settings. The pCC1FOS sequence (GenBank accession no. EU140751.1) was manually subtracted from resultant contigs in Geneious 8.1.9. The smallest cosmid insert was 28,221 bp, and the largest was 38,394 bp. The three contigs/cosmid inserts obtained for each strain were assembled to produce finalized sequences 48,228 and 30,236 bp in length for HS31 and PM13, respectively.

**FIG 1:**
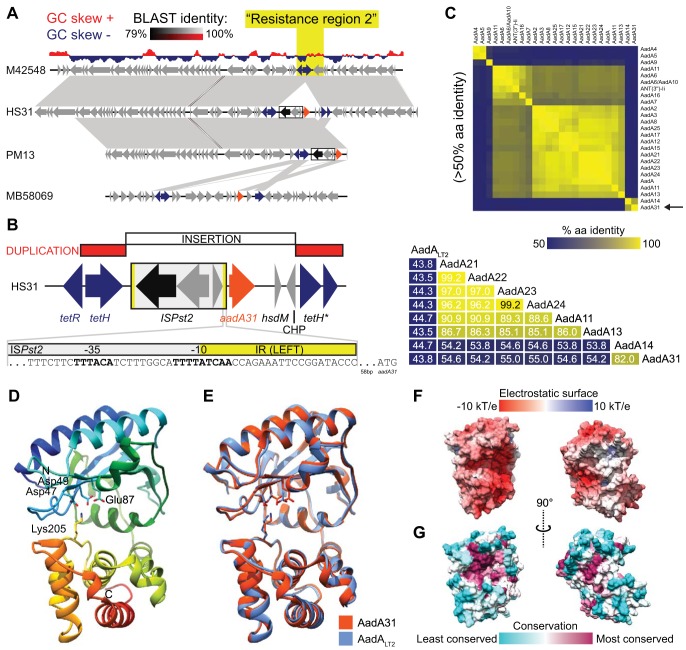
Genomic context, sequence identity, and structural homology modeling of the *aadA31* gene detected in *P. multocida* PM13 and *H. somni* HS31. (A) Genomic BLAST comparison of the PM13 and HS31 cosmid sequences, highlighting the insertion containing *aadA31*. The *aadA31* gene is absent from *M. haemolytica* M42548 but resides with the IS*Pst*2 element in a variant region syntenous with ICE*Mh1*. The *aadA31* gene is also present in the genome of *M. bovoculi* MB58069. (B) Gene context schematic of the insertion, depicting the novel insertion sequence from HS31 and the *tetR*-*tetH* duplication (truncated tetH* is represented as two arrows; truncated *tetR* is not shown). An expanded view of the IS*Pst*2 left terminal inverted repeat (IR) showing the putative promoter for *aadA31* (−35 and −10 boxes in bold) is also presented. IS*Pst*2 is inversely oriented such that the left inverted repeat is 58 bp from the *aadA31* start codon. (C) Predicted amino acid (aa) sequence pairwise identity matrix of *aadA* homologues (only those with >50% amino acid sequence identity in the CARD database). Shown at the bottom are the percent amino acid sequence identity values of selected AadA enzymes. (D) Predicted RobettaCM (21) ribbon model of the overall structure of AadA31. The N terminus is blue, and the C terminus is red. Conserved active site residues (20) are represented by sticks. (E) Superposition of the structures of AadA31 (orange) and AadA_LT2_ (light blue) (F) Electrostatic surface potential of AadA31 (showing orientations 90° apart). The highest electropositive and electronegative surfaces are blue and red, respectively. (G) Surface conservation plotted by ConSurf (22). The highest degree of conservation is magenta, and the lowest is cyan. All of the images are in the same orientation, except where indicated. Electrostatic surface potential was calculated with the APBS plugin and visualized in Chimera UCSF (23).

The HS31 and PM13 sequences shared 87.9% pairwise identity (MUSCLE alignment) and shared synteny with the integrative and conjugative element (ICE) MGE of *M. haemolytica* strain M42548 (GenBank accession no. CP005383) (Fig. 1A), which was isolated from a BRD case in Pennsylvania (15). Annotation identified a 789-bp open reading frame designated adenylyltransferase (ANT1), which was further identified as a streptomycin-3″-adenylyltransferase via a conserved-domain BLAST search. BLAST searches against the NCBI nonredundant (nr) database showed that the gene identified had been detected yet had not been characterized. We designated the gene *aadA31*—to the best of our knowledge, the lowest unassigned number.

Two instances of *aadA31* with 100% nucleotide identity were detected by a BLAST search of the nr database (i) in the draft genome sequence of *M. haemolytica* MhSwine2000, an isolate from the pneumonic lung of a juvenile pig in an Iowa grower house (M. J. Hauglund et al., USDA, unpublished data; GenBank accession no. ATTA00000000.1), and (ii) in the whole-genome sequence of *Moraxella bovoculi* strain Mb58069 (GenBank accession no. CP011374), an isolate from a case of infectious bovine keratoconjunctivitis in a yearling crossbred steer in Nebraska (16).

In PM13 and HS31, *aadA31* was adjacent to a 2,985-bp three-gene insertion (IS) element with 97% nucleotide sequence identity to IS*Pst*2, a widespread IS element identified in *Pseudomonas stutzeri* strain OX1 (17) (Fig. 1B). Discrimination of the element’s imperfect terminal inverted repeats (5′ GGGTATMCGGAWTTWMTGGTTGAT 3′) indicated that *aadA31* was harbored downstream from IS*Pst*2. Although the 58-bp sequence upstream of the *aadA31* start codon is conserved among PM13, HS31, and Mb58069, it does not appear to contain a promoter. Instead, putative −10 (TTTTATCAA) and −35 (TTTACA) boxes are partially located in the IS*Pst2* terminal repeat. Downstream from *aadA31* are genes for a putative type I restriction enzyme (*hsdM*; truncated) and a conserved hypothetical protein (CHP). Together, this sequence is a variation of syntenous resistance region 2 of ICE*Mh1* (15). Interestingly, these features reside within an insertion in *tetR*, which appears to have duplicated part of *tetR* and the *tetH* sequence, resulting in two copies that are either conventional or truncated (Fig. 1B). This suggests that resistance region 2 of ICE*Mh1* has a history of recombination.

We also tested for *aadA31* in a larger in-house collection of BRD agents isolated from feedlot cattle that died of BRD. Standard PCR amplification was performed in 20-μl reaction mixtures (HotStarTaq Plus master mix; Qiagen) containing 1 μl of heat-lysed bacteria in TE (10 mM Tris-HCl, 1 mM EDTA) and 0.5 μM oligonucleotides (ANT1_F, 5′ ATGACTACTAAACTAGATACCATAT 3′; ANT1_R, 5′ CTATTGCAGCTTCGTCGTC 3′; Eurofins Genomics). For positive controls, PCR was also performed with standard 16S oligonucleotides 27F and 1492R (18). The *aadA31* gene was detected in 40/42 *P. multocida* isolates and 5/5 *H. somni* isolates.

The closest relative of AadA31 is AadA14 (82% amino acid sequence identity) (Fig. 1C), which was first identified as plasmid borne in a bovine *P. multocida* isolate from Belgium (7). The next closest relative of both AadA31 and AadA14 is AadA23 from *Salmonella enterica* serovar Agona, which has 55.0 and 54.6% amino acid sequence identity, respectively (19). To provide further evidence that AadA31 is an AadA enzyme, we performed structural homology comparisons against the structure of AadA_LT2_ from *S. enterica* serovar Typhimurium LT2 (PDB code 4cs6) (20) with RobettaCM (21). AadA31 shares 43.8% amino acid sequence identity with AadA_LT2_. Least-squares superposition indicated that the predicted structure of AadA31 was consistent with the structure of AadA_LT2_ (root mean square deviation of 0.42 Å over 252 residues). Consurf (22) was used to calculate that the positioning of residues implicated in ligand binding and catalysis (20) was conserved within the active site, a highly electronegative cleft (Fig. 1D to G) (23).

To confirm that *aadA31* confers spectinomycin resistance and to assess resistance to other aminoglycosides, promoterless *aadA31* was cloned into *E. coli* under the control of the *lac* promoter of high-copy-number blue-white screening vector pGEM-T (Promega). *Taq*-based PCR amplification was performed as before with purified HS31 cosmid DNA in lieu of heat-lysed extractions. The amplicon was purified (Zymo DNA Clean and Concentrator kit) and ligated overnight at 16°C to pGEM-T in a standard 15-μl T4 DNA ligase reaction mixture (New England Biolabs). Ligated plasmids were transformed into chemically competent *E. coli* DH5α (Invitrogen) and plated on LB agar supplemented with isopropyl-β-d-thiogalactopyranoside (IPTG) at 0.5 mM, 5-bromo-4-chloro-3-indolyl-β-d-galactopyranoside (X-Gal) at 20 μg/ml, and ampicillin at 100 µg/ml (Fisher Scientific). Following overnight growth at 37°C, a white insert-containing colony was selected for susceptibility testing via the microtiter broth dilution method for MIC determination (24). The drug concentration range was 512 to 0.25 µg/ml, tested in triplicate in 96-well microtiter plates (Greiner CELLSTAR) with Mueller-Hinton broth (BD Difco). The MIC was the lowest concentration inhibiting visually detectable growth. Compared to the empty-vector control, the pGEM-*aadA31*-carrying strain exhibited resistance to spectinomycin (MIC, >512 µg/ml) and streptomycin (MIC, 256 µg/ml) but not to kanamycin, amikacin, or gentamicin (each MIC, 0.5 µg/ml). To demonstrate horizontal transfer of the ICE*Mh1* variant and to assess the spectinomycin/streptomycin MIC for *aadA31* under native promoter control in *E. coli*, *P. multocida* strain PM13 was mated with recipient rifampin-resistant *E. coli* DH5α as previously described (15). Transconjugants were selected on LB agar supplemented with rifampin (25 µg/ml; Millipore Sigma) and spectinomycin (50 µg/ml). Transconjugants were confirmed by PCR for *aadA31* as described above and with the UAL754 and UAR900 oligonucleotides for the *E. coli*-specific *uidA* gene (25). The spectinomycin and streptomycin MICs for a PM13-*E. coli* transconjugant versus empty-vector DH5α were >512 and 256 µg/ml versus 4 and 2 µg/ml, respectively. This was consistent with high-level (7) resistance activity of AadA class AMEs (6).

In conclusion, we identified a new *aad*A gene variant via functional screening of genomic cosmid-based libraries. Our investigation provides a striking example of the spread of ARGs; in this case, emerging in multiple bacterial species associated with bovine and swine respiratory disease and bovine keratoconjunctivitis. The results of this study also hint at the ongoing recombination of ARGs into and from MGEs such as ICE*Mh*1, a finding consistent with another published study (7) that also showed that MDR members of the family *Pasteurellaceae* can carry ARGs whose sequences have diverged considerably from those of homologues in other bacteria. These findings also highlight the interconnectedness of ARG transmission in MDR production animal and zoonotic pathogens and suggest that antimicrobial usage elsewhere in the production spectrum may impact the development and dissemination of resistant pathogens in distant animals.

### Accession number(s).

The *aadA31*, PM13, and HS31 cosmid DNA sequences obtained in this study have been deposited in GenBank under accession numbers MG520668, MG520669, and MG520670, respectively.
